# Retained Urethral Catheter Secondary to Placement in Proximal Ureter

**DOI:** 10.1155/2016/9178645

**Published:** 2016-04-10

**Authors:** Thomas B. McGregor, Rajan Sharda

**Affiliations:** ^1^Section of Urology, Department of Surgery, University of Manitoba, Winnipeg, MB, Canada R2H 2A6; ^2^Division of Urology, Department of Surgery, Cornwall Community Hospital, Cornwall, ON, Canada K6H 5S5

## Abstract

We present an unusual complication secondary to indwelling urethral catheter placement. Routine catheter placement by the obstetrics team in a postpartum female leads to retention of the catheter and inability of its removal by both the obstetrics and urology teams. Although a retained urinary catheter is relatively common, inability to remove a catheter secondary to placement inadvertently into a ureter is extremely rare. In this paper we will discuss the options in removing a retained catheter and present our case of a retained catheter secondary to placement within the right proximal ureter.

## 1. Introduction

Aberrant placement of Foley catheters within a ureter has been rarely reported in the literature [1–6]. To our knowledge a retained catheter secondary to this aberrant placement has only been reported once before [2]. The most common factor in these reports is a history of hydroureteronephrosis. In this case we present a retained Foley catheter secondary to aberrant placement within the ureter in a female postpartum with a history of physiologic hydronephrosis.

## 2. Case

A 28-year-old female underwent a spontaneous term vaginal delivery under epidural that was complicated by a minor episiotomy. After repair of episiotomy a 14 Fr Foley catheter was placed with inflation of the 10 cc balloon and then left to straight drainage. Following placement of the Foley catheter, no actual drainage of urine occurred through the Foley catheter. Instead, over the next couple of hours, urine promptly and continuously drained around the Foley catheter out of the urethra. At this point repositioning and/or removal of the Foley catheter was attempted and was unsuccessful. Nursing and housestaff were unable to deflate the Foley balloon and the Foley catheter was cut approximately 7 cm from the urethral meatus. Again no efflux from the balloon lumen was noted. Increased traction on catheter did not help and was poorly tolerated. The Foley catheter also drained poorly and was able to be flushed, but there was difficulty aspirating. Urology was consulted to aid in removal of the retained Foley catheter. On examination the labia were extremely swollen and tender from recent vaginal delivery and episiotomy. No Foley balloon was palpable on the anterior wall of vagina on vaginal exam. Several failed attempts were made to pass a wire down the balloon port to “pop” the balloon. Further examination and manipulation of the catheter were poorly tolerated and the patient was consented to undergo an exam under anesthesia and cystoscopy.

**Figure 1:**
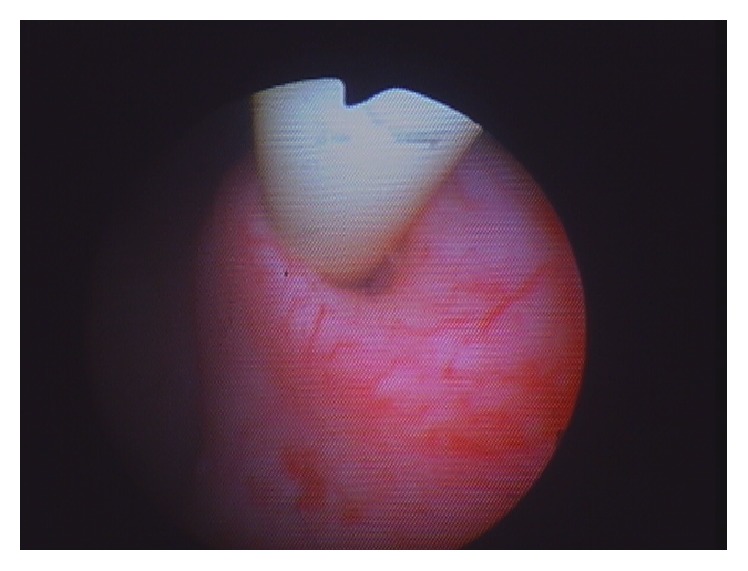
Cystoscopic view of Foley catheter extending through the ureteric orifice and up the ureter.

**Figure 2:**
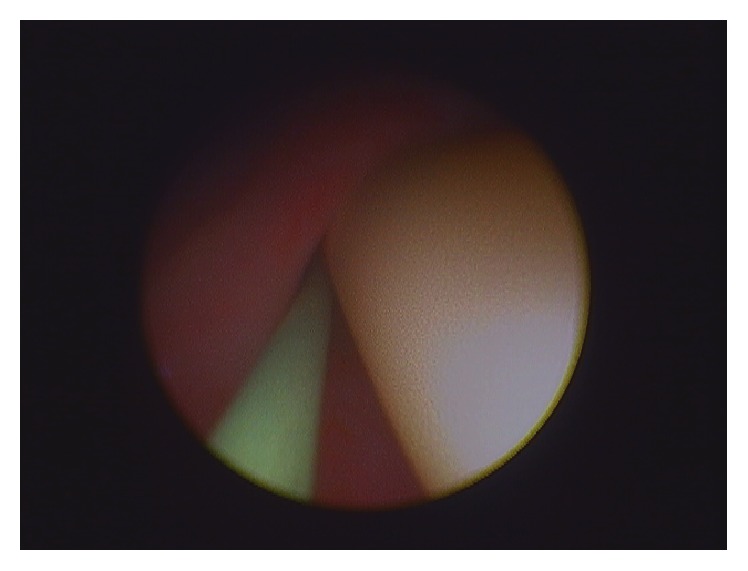
Guide wire placed alongside Foley catheter to facilitate passage of flexible ureteroscope.

**Figure 3:**
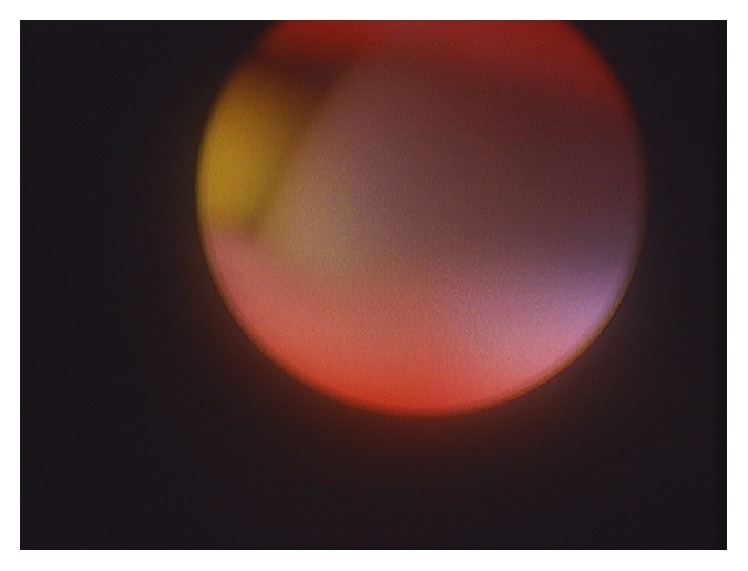
Foley balloon residing in proximal ureter, visualized through flexible ureteroscope.

**Figure 4:**
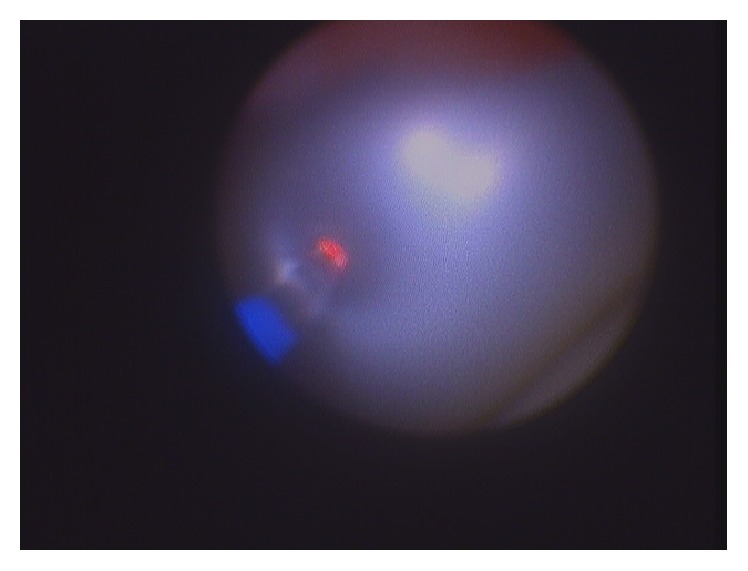
Laser fibre used to burst Foley balloon to facilitate removal of catheter from the ureter.

The patient was placed under conscious sedation and vaginal exam was carried out. The Foley catheter could be palpated within the distal bladder and urethra and was deviating to the right. No Foley balloon was palpable and the catheter was resistant to traction. Cystoscopy was performed alongside the retained Foley. Cystoscopy revealed the Foley catheter intubating the right ureteric orifice and traveling upwards in the ureter (Figure 1). A ureteric catheter was placed alongside the Foley catheter and a retrograde pyelogram was performed (Figure 2). Retrograde pyelography showed the Foley balloon within the proximal ureter with only a minimal amount of contrast passing proximally. A hydrophilic guide wire was able to be passed alongside the Foley catheter to the level of the kidney with some difficulty. Ureteroscopy was then carried out with a semirigid ureteroscope and revealed a Foley catheter kinked upon itself with balloon fully inflated in the proximal ureter (Figure 3). A hole was created in the balloon with the use of HO:YAG laser (Figure 4). As the balloon began to deflate the catheter unkinked and the previously placed fluid in the balloon began to drain from the catheter balloon port lumen (see Video 1 of the Supplementary Material available online at http://dx.doi.org/10.1155/2016/9178645). The catheter was then easily removed. A ureteric stent was then placed on the right side to allow healing and prevent ureteric obstruction secondary to edema. Ultrasound confirmed the appropriate placement of the right ureteric stent and noted mild hydroureteronephrosis on the right. The patient was discharged home postpartum day 2 and was seen in follow-up for stent removal 2 weeks later without any residual effects.

## 3. Discussion

Placement of an indwelling urinary catheter is a very common procedure and although the complication of a retained catheter is relatively rare in comparison to the amount inserted, this still represents a common problem faced by urologist. A Foley catheter balloon is usually inflated with 10 cc of sterile water to maintain the catheter in place. To insure that the catheter is in the correct location, one can consider inflating the balloon with 3 cc of water and then pulling the balloon down to the bladder neck prior to completing the rest of the 10 cc inflation. Following this, observation of urine drainage from the catheter shows that the catheter is in the correct location.

When difficulty arises in removing a catheter this is usually due to the inability of the balloon to deflate. Commonly, the balloon port is cut off in an attempt to deflate the balloon passively [7]. This circumvents any defect in the inflation port itself. Some have advocated injecting fluid within the balloon port until the balloon burst [8] or using solutions such as mineral oil to dissolve the balloon [9]. These techniques require inspection of the balloon to ensure that there are no retained particles. The use of guide wires down the inflation port to burst the balloon has also been utilized [10]. Using needles with a palpable balloon through the urethra or vagina, or if needed under ultrasound guidance, is often a final resort [7].

In this case we show the failure of several of these techniques largely due to the fact that catheter was found folded upon itself within the ureter, yielding the Foley balloon nonpalpable. This did not allow for any fluids or a guide wire to be passed through the inflation port. The ability to perform a proper vaginal exam and to pass a cystoscopy alongside the retained Foley allowed for easy diagnosis and ureteroscopic management. A handful of cases have reported aberrant placement of a urethral catheter within the ureter, but only one of those catheters was retained. We believe this to be the second case reporting a retained urethral catheter secondary to placement within the ureter.

## 4. Conclusion

Aberrant placement of urethral catheter into a ureter is extremely uncommon, and having a catheter becoming retained in that position is even more exceedingly rare. The findings in this case show that unusual complications can occur in the most common of procedures. We have described here a viable option for managing a retained Foley balloon in a proximal ureter with the use of ureteroscopy and laser. Perhaps the length of Foley catheter remaining outside of the urethral meatus should be used as guide for proper placement of a catheter within the bladder. In patients with a history of hydroureteronephrosis, as is common in pregnant or postpartum patients, the possibility of placement within the ureter is a real possibility. One should avoid overzealous placement of Foley catheter length into the bladder to avoid the possibility of more proximal migration of the Foley into a dilated ureter in this patient population.

## Supplementary Material

This video demonstrates the end-urologic management of a retained foley catheter in the proximal ureter of a patient. You can see the ureteroscopic view of the foley balloon in the proximal ureter. A 200 micron laser fiber is used to burst the balloon, facilitating it's removal.
